# Licking County Medical and Philosophical Society

**Published:** 1840-11

**Authors:** 


### LICKING COUNTY MEDICAL AND PHILOSPHICAL SOCIETY.

We lately had the pleasure of attending one of the meetings of a new scientific association at Newark, Ohio, under the above title; and are indebted to Dr. Marble, one of its members, for an account of its organization and objects. In contemplates the cultivation of mineralogy, geology, chemistry and Botany, in connexion with medicine, and provides for admitting as members, gentlemen who are not of the medical profession. It looks to the establishment of a museum, the delivery of lectures and essays, and the gradual formation of a library. Some of the members have a number of specimens of pathological anatomy and comparative anatomy which will be made the basis of a cabinet of that kind. It is expected to have courses of lectures to which the community at large will be admitted.

The Society consists of four classes of members. 1st. *Senior members*, consisting exclusively of physicians. 2d. *Junior members*, consisting of students of medicine. 3d. *Active honorary members*, residing near enough to participate in the regular meetings of the Society. 4th. *Honorary members*, residing at a distance.

The Society elects its officers annually and holds regular quarterly meetings. The following are the officers, for the present year:

John I. Brice, *President;* Daniel Marble, *Vice President;* Dr. J. N. M'Millan, *Recording Secretary;* A. O. Blair, *Treasurer;* D. Marble and J. Dille, *Corresponding Committee;* Edward Stansberry, John M. Wilson, D. Marble, A. O. Blair, E. F. Bryan, *Censors;* F. B. Parmele, V. H. Roe, A. D. Bigelow, and J. Dille, *Curators.*

We wish our Newark friends, in this praiseworthy effort to promote the cultivation not only of medicine but its associate sciences.

